# The role of self-efficacy, entrepreneurial passion, and creativity in developing entrepreneurial intentions

**DOI:** 10.3389/fpsyg.2023.1134618

**Published:** 2023-03-06

**Authors:** Macário Neri Ferreira-Neto, Jessyca Lages de Carvalho Castro, José Milton de Sousa-Filho, Bruno de Souza Lessa

**Affiliations:** Post-graduation Program in Management, University of Fortaleza, Fortaleza, Ceará, Brazil

**Keywords:** entrepreneurship, entrepreneurial intention, creativity, entrepreneurial passion, self-efficacy

## Abstract

Although studies aimed at understanding entrepreneurship have analyzed passion, creativity, and entrepreneurial self-efficacy, few studies include these antecedents in the same model. In this sense, this study aims to assess the relationship between passion, self-efficacy, and creativity with entrepreneurial intention. The data was collected through a survey and the questionnaires were applied to university students who formed a sample of 190 respondents, and such data was analyzed using structural equation modeling based on partial least square technique. Regarding our results, the relationship between creativity and entrepreneurial intention has not been confirmed. The multigroup analysis revealed that the level of education influences men’s entrepreneurial intention and creativity only influence entrepreneurial intention when mediated by entrepreneurial passion. This study contributes by highlighting the roles of analyzed passion, creativity, and entrepreneurial self-efficacy in entrepreneurs from one of the largest emerging economies in the world. Moreover, it also contributes to academia as it confirms the explanatory power of the Theory of Planned Behavior as a tool to understand the cognitive foundations of entrepreneurship. It also offers a practical contribution by signaling to public policymakers which features should be incentivized to boost entrepreneurship in emerging economies.

## 1. Introduction

Entrepreneurship has been pointed out as key for economic development, as it generates income and jobs for the populations in different contexts (Zhao et al., 2005). As it is intricately connected to entrepreneurship, entrepreneurial intentions have become a widely studied topic, as research has been dedicated to better understand the antecedents and consequences of entrepreneurship as a social phenomenon (Ferreira et al., 2017).

In this sense, several authors have demonstrated the factors leading individuals to become entrepreneurs, including self-efficacy, entrepreneurial passion, and creativity (Ajzen, 1991; Shane et al., 2003; Chen et al., 2009; Drnovšek et al., 2010; Engle et al., 2010). Moreover, research has revealed the existence of a relationship between creativity, self-efficacy and entrepreneurial passion (inventor) and entrepreneurial intentions (Chen et al., 1998; Drnovšek et al., 2010; Moralista and Delariarte, 2014; Campos, 2016), which allows one to have a positive perspective of scientific work in the area.

Nonetheless, research on entrepreneurial passion is still in its academic infancy, especially in the context of the relationship between passion and creativity (Chen et al., 2015). For instance, there are few models in the literature encompassing passion, self-efficacy, and creativity as antecedents of entrepreneurial intentions and, to address to this gap, this study explores the direct relationship between these constructs. Furthermore, the understanding of how such a feeling influences entrepreneurial intentions and related activities is vital to fomenting actions aiming to boost economic development at different levels, since entrepreneurial activity has been recognized as key for it.

Furthermore, the choice for passion and creativity was also underpinned by recent research advances. As such, this study contemplates, i.e., that creativity positively influences entrepreneurial intention whereas passion partially mediates the relationship between creativity and intention (Murad et al., 2021). A discussion that has been confirmed in different contexts and brings forward the fact that entrepreneurial passion positively affects entrepreneurial intention and entrepreneurial self-efficacy (Neneh, 2022).

On the one hand, it is understood here that passion is key to beginning a business venture since it stimulates motivation, enhances intellectual activity, and gives meaning for daily work. Especially among potential entrepreneurs like university students, individuals who might start their businesses, for instance, as a way to make a living after finishing their studies (Anjum et al., 2021). On the other hand, creativity was also chosen as it has been demonstrated that people’s creativity can mediate the relationship between their perceptions that societal norms do not support their entrepreneurial intentions, for example (Ng and Clercq, 2021). We thus consider these two elements influence key elements underlying intentions (Kiani et al., 2022).

Considering these aspects, this study addresses to the following research problem: what is the impact of self-efficacy, passion, and creativity as antecedents of entrepreneurial intention? Thus, this study aimed to analyze the relationship between entrepreneurial passion, creativity, and self-efficacy predictors of entrepreneurial intention. To achieve these research objectives, questionnaires were applied to 190 individuals, and the data obtained were treated with descriptive statistics and analyzed by employing structural equation modeling through the Partial Least Square technique. In this sense, this study contributes to scholarship by providing a model able to explain entrepreneurial intentions based on three important characteristics of individuals, namely, passion, creativity, and self-efficacy.

Accordingly, this paper contributes to scholarship by advancing the analyses of how feelings such as passion and creativity influence entrepreneurial intentions, which, in their turn, impact entrepreneurial activities. Moreover, there is also a practical contribution as universities and private companies can take advantage of these analyses to elaborate or reform curricula and processes, focusing on the impact the mentioned feelings have on potential entrepreneurs. In addition, public policymakers may use these conclusion to deliberate public policies oriented toward incentivizing entrepreneurial activity.

Moreover, this study contributes to entrepreneurship theory and to the cognitive foundations of entrepreneurship by demonstrating the influence of psychological factors such as self-efficacy, entrepreneurial passion, and creativity and their impacts on entrepreneurial intentions. Thus, this research contributes to these theories by demonstrating the roles these elements have as they influence and interact with intentions.

For organizing purposes, this paper is divided into six sections. After this Section “1. Introduction,” the next Section “2. Theoretical backgrounds” deals with the theoretical backgrounds underpinning this paper. Afterwards, the third Section “3. Methodology” presents the methodology used to operationalize this research. The fourth Section “4. Results” then introduces the results, and it is followed by the fifth Section “5. Discussion,” in which those results are discussed. Finally, the sixth Section “6. Conclusion” displays the conclusion reached here.

## 2. Theoretical backgrounds

### 2.1. The theory of planned behavior

The willingness to start a business venture can be defined more as a planned behavior rather than an improvised decision, taking into account the number of elements comprised in taking such a decision (Krueger et al., 2000). Hence, the TPB is an essential theory to explain entrepreneurial intention (Al-Jubari, 2019; Duong et al., 2020; Lopes et al., 2020). Ajzen (1991) presented the TPB using it to investigate the factors impacting intention and to predict them, feature making it useful for this research as well. Futhermore, the TPB supplies an appropriate theoretical lens to study entrepreneurial intention deeming individual and social factors concomitantly (Liñán and Chen, 2009).

Accordingly, the TPB considers that three variables influence entrepreneurial intentions more directly. First, there is perceived behavioral control (PBC, that is, individuals assessing a to-be-executed behavior according to its ease of execution), attitude toward entrepreneurship (personal belief in specific behaviors or actions), and subjective norms [an individual’s perceptions of what people around them or relevant others think about a specific behavior (starting business ventures, for instance)] (Ajzen, 1991; Liñán et al., 2016; Al-Jubari et al., 2019).

Therefore, intention can be defined as an individual’s mental focus to achieve a predetermined goal. In this sense, operationalizing a business idea is preceded by the desire to do so (Bird, 1988), and when this specific kind of intention takes place, it is then referred to as entrepreneurial intention (Davidsson, 1995). Although there has been an increasing number of publications on the role of intentions in the entrepreneurial process (Liñán and Fayolle, 2015), there is still a gap in research on how to enhance the presence of higher education students in entrepreneurial activities so they can tackle the problems of a globalized world (de Alencar and Fleith, 2010; Rosairo and Potts, 2016).

### 2.2. Entrepreneurial intention

Entrepreneurial intention refers to an individual’s willingness to start a new venture (Engle et al., 2010). In the TPB, intention is the central factor in indicating how much individuals intend and plan to engage in a behavior (Ajzen, 1991). Intention is a key antecedent of action; thus, the study of entrepreneurial intention deepens the understanding of entrepreneurial knowledge and behavior patterns. Intentions are also the result of the interaction between individuals and their context, and their analysis focuses on their influencing factors (Sun et al., 2011).

In this sense, intention can be described as the mental interpretation of the actions necessary to establish new independent businesses or to create value for existing companies (Fini et al., 2012). Thompson (2009) defines intention as the certainty a person who wants to open a business has and consciously plans to do it in the future. In the same sense, entrepreneurial intentions are factors motivating and influencing people searching for entrepreneurial results (Hisrich et al., 2014).

Bearing that in mind, the main individual predictors for intentions are personal traits, motivations for private fulfillment, positive perspective, self-efficacy, perception of management, locus of control, perception of barriers, and creative thinking (Ferreira et al., 2017).

This study aligns itself with others which studied similar samples, namely, university students as emerging entrepreneurial subjects, it also employed the theory of planned behavior, attesting its explanatory power. Moreover, i.e., focusing on self-efficacy, it shows its effects on entrepreneurial intentions and demonstrates the potential roles of entrepreneurial education on the willingness of individuals to start their own ventures (Liu et al., 2019; Lv et al., 2021).

Such kind of education can influence not only traditional entrepreneurship, but also social entrepreneurship, which can also take advantage of TPB to advance a solid research agenda in the same way to what was carried out here (Chien-Chi et al., 2020). Furthermore, besides self-efficacy, this study also brings into light variables such as passion and creativity, a specificity that makes it as interesting as other studies that analyzed interesting psychological elements like narcissism, psychopathy, and Machiavellianism and the influence of those traits on entrepreneurial intentions, for instance (Wu et al., 2019).

### 2.3. Creativity

Creativity might be described as the imagination to “invent” something new and valuable, transforming an already existing feature into something better (Young, 1985). Furthermore, it can be defined as the generation of new and adequate solutions to problems that need them in any domain of human activity (Amabile, 1997). Creativity also manifests itself in the form of surprising, unique results, (Puhakka, 2012) involving three components: skills, novelty, and value (Young, 1985).

In the same sense, creativity is the ability to produce new and suitable things according to social reality, which requires from the entrepreneur a context and related processes, as well as the interaction between these elements so that she or he can produce such novelties and generate business opportunities. Following this perspective, creative thinking is vital for entrepreneurial behavior as it enables the identification of opportunities further connected to long-living organizations (Sternberg and Lubart, 1999; Ko and Butler, 2007; Puhakka, 2012).

Therefore, entrepreneurial creativity transcends ordinary creativity, starting from the perception of an opportunity bringing financial gains. In addition, such perception involves the definition of a problem, the generation of ideas, and the implementation of these new ideas, which impel the generation of new products, services, or processes (Amabile, 1997; Puhakka, 2012; Smith et al., 2016). In other words, creativity is a process through which inventions occur; it is how new things are created.

In this context, creativity also underscores the resourceful capacity to bring new features into existence, i.e., making something new, which leads to motivation and differentiates regular products and services from disruptive ones (Sternberg and Lubart, 1999; Okpara, 2007). However, creativity also works along with other abilities such as the flexibility to cope with changes, the capacity of playing with concepts and possibilities, a flexible perspective for dealing with things, and the habit of appreciating the present while searching for ways to improve it (Smith et al., 2016).

In addition, an entrepreneur normally needs to make decisions influenced by the organization’s resources. Nonetheless, it is common for entrepreneurs to make impactful decisions regardless of the resources available, based much more on their own intuition. In this sense, the entrepreneur must demonstrate strong leadership, shaping business strategy and motivating employees through creative thinking (Fillis and Rentschler, 2010). Likewise, individuals with ideas for starting businesses are more likely to have viable perceptions about the recognition of opportunities and, thus, tend to have greater entrepreneurial intentions (Okpara, 2007).

### 2.4. Self-efficacy

Bandura (1977) defined the concept of “self-efficacy” as the origin of an individual’s skill to complete a specific task and perform a job. It relates to how actions, behavior, perceptions, cognition, and the environment influence each other in a self-motivated way (Shahab et al., 2019). Self-efficacy is also outlined as people’s beliefs about their abilities to fulfill expected levels of performance, influencing events with an effect on their lives (Bandura, 1994). The perceived self-efficacy not only defines the range of options to be considered but also affects other aspects of decision-making. Making decisions in no way ensures that the necessary courses of action be successfully implemented, thus, self-efficacy refers to beliefs about what one can do, and the expectations of results indicate the expected consequences of what might be accomplished (Ajzen, 1991; Bandura, 2001; Schwarz et al., 2009; Drnovšek et al., 2010; Shahab et al., 2019).

In this perspective, entrepreneurial self-efficacy can include objective beliefs, meaning the ability to assess whether an individual can successfully engage in activities, and control beliefs, implying the capacity to manage negative and positive thoughts while pursuing goals (Drnovšek et al., 2010). Furthermore, people’s beliefs in their effectiveness influence the kind of situations they are able to plan, build and operate. Those with a high sense of effectiveness read situations of success, which offer positive guidelines and support for performance (Bandura, 1993). In other words, self-efficacy can be considered a sort of task-specific self-confidence (Shane et al., 2003). In this perspective, human behavior is highly influenced by the belief in their ability to perform the set of behaviors necessary to succeed, demonstrating a strong relationship between self-efficacy and behavior (Engle et al., 2010).

The theory of planned behavior (TPB) places belief in self-perceived behavioral control or efficacy within the more general structure of the relationships between beliefs, attitudes, intentions, and behavior (Ajzen, 1991). In the same sense, the three antecedents of intention are personal attitude toward behavioral results, subjective norms, and perceived behavioral control (self-efficacy) (Schwarz et al., 2009). Perceived behavioral control refers to individuals’ perception of the difficulty to perform an activity and, in this sense, self-efficacy and perceived behavioral control are constructs analogous to each other.

Moreover, perceived behavioral control and self-efficacy are similar since they are involved with the perceived ability to perform a behavior (or sequence of behaviors) (Ajzen, 2002). In addition, an individual with high self-efficacy for a given task tends to engage in more effort for a longer period, persist despite setbacks, set and accept higher goals, and develop better plans to accomplish them (Shane et al., 2003). Hence, the higher the belief one has in its abilities, the greater its entrepreneurial intentions (Moraes et al., 2018).

### 2.5. Entrepreneurial passion

Entrepreneurial passion means a significant emotional state for entrepreneurs. Along with cognition and behavioral expression of high personal value, it is a powerful indicator of entrepreneurs’ enthusiasm for setting up businesses (Chen et al., 2009). Entrepreneurial passion can also be defined as a series of complex patterns of mental, brain, and physical reactions, activated and maintained by enthusiasm, thus, entrepreneurial passion can be considered a central element of entrepreneurial efforts (Cardon et al., 2009).

Moreover, entrepreneurial passion motivates entrepreneurs to recognize opportunities and create new businesses, being regarded as an important part of business motivation and success (Shane et al., 2003; Cardon et al., 2009). However, this kind of passion is not a personality trait, but an internal emotional state that an individual lives while thinking about or participating in entrepreneurial-related activities (Cardon et al., 2009).

Passion is also required as a means to accomplish high levels of performance and overcome barriers, and it can also lead entrepreneurs to relevant business results. In this regard, Carden et al. (2009) identified three role identities (inventor identity, founder identity, and developer identity) in distinct aspects of the processes related to different types of passion. First, the inventor status happens when entrepreneurs are passionate about activities focused on identifying, inventing, and exploring new opportunities. Second, the founder’s identity relates to entrepreneurs’ passion for activities aimed at starting businesses to explore new market opportunities. Third, the developer status takes place is linked with the entrepreneurs’ passion for activities related to growing, developing, and expanding their business. While each of these roles may operate independently of one another, some entrepreneurs may be passionate about all of these identities, while others may think one identity is more important than the others.

These different identity-related passions can affect goal-related perceptions and produce specific business outcomes (Cardon et al., 2009). Therefore, entrepreneurial passion acting through its elements of strong positive emotions associated with important identities is a key motivating factor for entrepreneurial behavior, especially when resources are limited, and the environment is uncertain (Chen et al., 2009; Huyghe et al., 2016).

In other words, entrepreneurial passion includes deep and consciously positive feelings, vital to personal identity. The combination of these two aspects (strong positive emotions and identity centrality) leads to lasting emotional experiences, which often last longer than emotional episodes. Thus, entrepreneurial passion can be theoretically defined and measured through the following dimensions: strong positive emotions and identity centrality, being reflected in three role identities: invention, foundation, and development (Cardon et al., 2013).

### 2.6. Theoretical model and hypotheses

**FIGURE 1:**
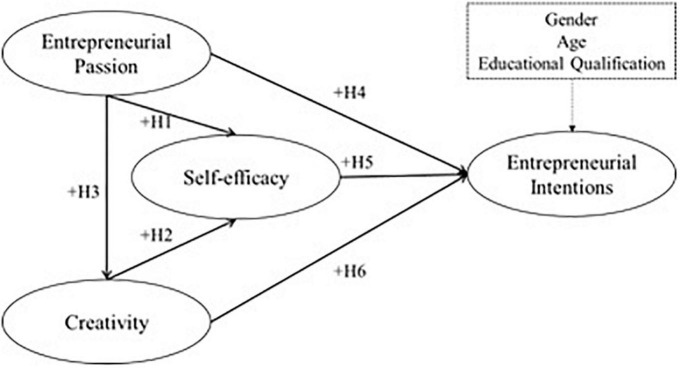
Theoretical model.

Considering its objectives, the model proposed here takes entrepreneurial intention as a dependent variable and three categories as predicting ones, namely, perceived self-efficacy (perceived behavioral control), entrepreneurial passion, and creativity. It is noteworthy this study analyzed the existing direct relationships; further indirect relationships were not in the model’s scope as they strayed from our research question and objectives. To make it clearer, Figure 1 depicts the model.

Cardon et al. (2009) identified entrepreneurial passion as a trigger for one to engage in entrepreneurial activities since individuals need to put together and develop sets of skills enabling them to become successful entrepreneurs. This is particularly important at the beginning of a venture when such skills are essential and should be developed in the simplest as well as swiftest way. In this regard, since these skills are boosted, self-efficacy levels increase, and individuals tend to become more aware of their success prospects.

Furthermore, passion affects entrepreneurs’ choices on the decisions they are supposed to make to accomplish their goals. This is a double-folded process with particular characteristics and results. On the one hand, passion might turn into an obsession, making it negatively difficult for individuals to realize the depth of problems they have and to believe falsely to hold the conditions needed to engage in certain business ventures. On the other hand, passion may drive entrepreneurs to feel the pleasure of what other people would consider an ordeal, that is, making it better for them to cope with internal and external pressures. Thus, entrepreneurs would feel more focused on processes and on improving themselves as well as their ventures’ performance, which has a closer relationship with the capacity of changing reality underlying the self-efficacy concept (Vallerand et al., 2007; Lafrenière et al., 2011; Stroe et al., 2018).

Accordingly, Baron (2008) believed self-efficacy modifies one’s cognitive operation and explained this perception depends on disruptive thinking and on outlining the probabilistic patterns leading one to pursue a career in entrepreneurship (Mannino and Faraci, 2017). In certain cases, new ideas often cause people to rethink their ability to innovate while starting a business passionately, thus, high passion levels contribute to having a greater perception on the possibilities of starting a lasting venture. Based on this argument, the first hypothesis goes as follows: H1–Entrepreneurial passion is positively related to self-efficacy.

Creativity can be firstly defined as being able to create new and value-added products or services (Amabile, 1996). In this study, it is also considered a combination of knowledge in people’s conscious minds, allowing them to reflect on how to develop new innovative and intelligent ideas (Chen et al., 2015). In this sense, creativity might be regarded as the most important feature for starting a company, as it is very useful to expand people’s prospects of how much success they may accomplish (Godfrey, 1996).

Creativity might be considered an intrinsic skill, being born in the individual and happening by chance to each person. Conversely, it can also be seen as a characteristic to be boosted and increased throughout one’s life. In this sense, creativity links to self-efficacy as creative people tend to be confident in a way represented, for example, by the belief they have the necessary tools to overcome problems, even when the context seems dire. In other words, creativity as a flexible attribute makes people challenge reality and see they are capable of doing more than what appears to be possible. In opposition, when individuals believe creativity is a static characteristic, they show smaller self-efficacy levels (Wood and Bandura, 1989; Tierney and Farmer, 2002; Royston and Reiter-Palmon, 2019).

Furthermore, in socio-cognitive theories such as TPB, self-efficacy refers to a motivational construct following the choice of activities, goal levels, and persistence, as well as the dynamics of business performance in different contexts (Drnovšek et al., 2010). In the literature on the antecedents of entrepreneurial intentions, self-efficacy is important because entrepreneurs must be confident in their ability to perform different and often unexpected tasks during situations of uncertainty (Bellò et al., 2018). Self-efficacy thus emerges as a crucial antecedent to the intention and its study is one of the main contributions in entrepreneurial intentions research (Chen et al., 1998; Drnovšek et al., 2010; Krueger, 2017). Nevertheless, researchers have not yet defined the role of entrepreneurial self-efficacy in the relationship between different types of antecedents and entrepreneurial behavior (Bellò et al., 2018), and such gap has led to the formulation of the following hypothesis: H2–Creativity is positively related to self-efficacy.

Considering the influence of passion on creativity, it is noteworthy that while the former leads individuals to engage fiercely in a venture, the latter enables them to persist as they become more aware of possibilities to overcome emerging obstacles. Furthermore, the connection between these two constructs may lead other people to feel motivated by what they see entrepreneurs planning, doing, and accomplishing, i.e., a person with high levels of passion and creativity is likely to affect other people to feel the same. Passionate entrepreneurs demonstrate to others the possibilities for challenging seemingly unchangeable norms (Hatfield et al., 1994; Chen et al., 2009; Cardon et al., 2013; Davis et al., 2017).

In addition, learning derived from past experiences might increase creativity levels, thus, recursively, and positively affecting passion as such learning can be employed through creative thinking and generate successful outcomes. In their turn, these outcomes are likely to exert a positive influence on others. In this regard, obstacles and challenging issues can start creative processes increasing entrepreneurial passion as new alternatives to solve problems are developed. Moreover, such solutions recursively improve self-perceptions about creativity, deepening the engagement in starting a business venture (Amabile, 1996; Zhou et al., 2012; Biraglia and Kadile, 2017).

Furthermore, there is a linear relationship between positive emotions and creativity, as the higher, a person’s level of positive emotions, the more creative their performance becomes, dynamics consistently affected by the environment where these relationships take place (Amabile et al., 2005; Baron, 2008). Correspondingly, entrepreneurial passion can be defined as the consistent and conscious positive emotion experienced by participating in entrepreneurial activities. In this sense, the passion for invention in particular affects problem solving, drives people to set new and creative courses of action, and being so, there is a significant relationship between passion for invention and creativity (Cardon et al., 2009, 2013). Following these arguments, a person’s passion for invention is likely to influence their creativity, which leads to the next hypothesis: H3–Entrepreneurial passion is positively related to creativity.

Entrepreneurial passion engenders both positive feelings and the identities related to them. This connection, which has been widely documented in the scholarly literature on the topic, plays an even more important role when there is little availability of resources and when the external context is uncertain (Cardon et al., 2009; Chen et al., 2009; Murnieks et al., 2014; Huyghe et al., 2016). In this sense, it is possible to state there is a clear relationship between entrepreneurial passion and entrepreneurial intention since the former is key for the latter by making entrepreneurs more capable of acknowledging opportunities and prone to start new ventures, thus, being an essential part of motivation and success for entrepreneurship (Shane et al., 2003; Carsrud and Malin, 2011; Karimi, 2020).

Furthermore, entrepreneurial passion is likely to be positively linked to intentions as it might increase personal commitment and energize people into finishing important tasks, especially when businesses are in their infancy. In other words, passion is also relevant for intent for its role in motivating entrepreneurs to keep up with their objectives and to commit them with what had been previously planned. In addition, entrepreneurial passion cannot be regarded as a static attribute since individuals can present different levels of desire for learning and questioning standardized norms or behaviors, another feature related to entrepreneurial intentions (Bierly et al., 2000; Cardon et al., 2013; Karimi, 2020; Syed et al., 2020).

Forming intention is also regarded as the first step in the process of creating a new business. Accordingly, entrepreneurial passion can help one to start plans to start a new enterprise since passion motivates entrepreneurial activity. Passionate stakeholders tend to have strong and positive feelings about the entrepreneurial activity they participate and have a consistent motivational drive to act on those feelings (Cardon et al., 2009; Nasiru et al., 2015; Biraglia and Kadile, 2017; Neneh, 2020). In this context, the fourth hypothesis is presented: H4–Entrepreneurial passion is positively related to entrepreneurial intention.

Entrepreneurial self-efficacy relates to the personal belief one has in his or her ability to carry out certain tasks, in this case, the ones concerning entrepreneurship. In this perspective, it can be also described as the degree of such belief, in the sense of how much a person deems to have the capabilities needed to engage in starting a business (Barakat et al., 2014; Alammari et al., 2019). As such, this kind of self-efficacy connects to entrepreneurial intentions as, for instance, high levels of it indicate a positive tendency to both addressing to the problems of launching a company and to put with the processes required to do so successfully (Hassan et al., 2020; Elnadi and Gheith, 2021).

In other words, self-efficacy comprises how confident individuals are in their abilities and skills associated with successfully tackling entrepreneurial activities. People with high levels of entrepreneurial self-efficacy tend to be bolder in overcoming obstacles than others with low levels of it (Bandura, 2000; McGee et al., 2009; Memon et al., 2019). Considering the risks and the initiative required to start a business, self-efficacy plays an important role in affecting entrepreneurial intentions as it fosters enthusiasm, commitment, and persistence, boosting the possibility to achieve entrepreneurial success (McGee and Peterson, 2019; Newman et al., 2019; Elnadi and Gheith, 2021).

Studies examining the direct impact of self-efficacy on entrepreneurial intentions revealed that people with higher self-efficacy have greater intentions, and also believe they are more likely to achieve positive outcomes by tracking a determined plan (Drnovšek et al., 2010). Moreover, entrepreneurs gauge their certainty in their talents based on self-efficacy and tend to be persistent as well as certain about their possibilities of success. In this perspective, there have been studies confirming that higher self-efficacy levels are positively related to higher entrepreneurial intentions (Chen et al., 1998; Cardon and Kirk, 2015). Bearing these elements in mind, the hypothesis below is proposed: H5–Self-efficacy is positively related to entrepreneurial intention.

In more general terms, creativity can be described as the ability to foster new ideas as well as strategies to recognize opportunities and solve problems. In this perspective, it might be associated with developing new ways to perform a task instead of performing it following a more usual methodology (Raposo et al., 2008; Zimmerer et al., 2008). In other words, creativity is related to discovering new forms of seeing things. Accordingly, creativity can be accounted as a key characteristic for entrepreneurs, contributing to higher levels of entrepreneurial intentions (Kusmintarti et al., 2014, 2017).

Moreover, creativity has been deemed as an essential cognitive tool for fostering proactive behaviors since it relates to individuals’ ability to overcome and surpass existing setbacks. It might also be argued that creativity is fundamental to increasing entrepreneurial intentions levels as it improves entrepreneurial orientation and allows greater opportunity recognition (Gilad, 1984; Puhakka, 2012). In this perspective, people with higher creativity levels respond better to problems, seek more information, and are more successful in avoiding stressors. Thus, creativity is likely to influence entrepreneurial intentions positively since it makes perceptions regarding entrepreneurship promising (Zampetakis et al., 2009; Lerch et al., 2015; Kusmintarti et al., 2017).

Correspondingly, creativity is normally related to creative and innovative business ideas, and it refers to the skills and resources people have in order to develop new and useful ideas. Accordingly, entrepreneurs are usually creative individuals, thus, creativity and creative thinking account as essential entrepreneurial characteristics and need to be taken into consideration in intention-based models, which is also the case in this study. It is also possible to state that the disposition for creativity can boost confidence and make one see greater success possibilities in starting a venture (Hamidi et al., 2008; Nasiru et al., 2015; Biraglia and Kadile, 2017; Murad et al., 2021). Thus, the sixth hypothesis goes as follows: H6–Creativity is positively related to entrepreneurial intention.

## 3. Methodology

As previously mentioned, this study aimed to analyze the relationship between entrepreneurial passion, creativity, and self-efficacy as predictors of entrepreneurial intention by employing questionnaires in a sample of university students (Cooper and Schindler, 2016). This non-probabilistic sample comprised 190 respondents, who were reached electronically through social networks over the course of 2021. For such, a seven-point Likert questionnaire with questions on sociodemographic characteristics, self-efficacy, creativity, entrepreneurial passion, and the entrepreneurial objective was used with this sample. Table 1 presents the constructs and its theoretical backgrounds.

**TABLE 1:** Constructs and items.

Construct	Question	References
Entrepreneurial passion (EP)	It is exciting to figure out new ways to solve unmet market needs that can be commercialized.	Cardon et al. (2013)
	Searching for new ideas for products/services to offer is enjoyable to me.	
	I am motivated to figure out how to make existing products/services better.	
	Scanning the environment for new opportunities really excites me.	
	Inventing new solutions to problems is an important part of who I am.	
Creativity (CR)	In everyday life, I find it easy to solve problems.	da Silva and Nakano (2019)
	I think of different ideas when I face a problem.	
	People say that I have different ideas.	
	I prefer to create new solutions than to use existing ones.	
	I prefer to look at a situation from different points of view.	
	I usually describe my ideas carefully and in detail.	
	I like activities in that can use my imagination.	
Perceived behavioral control (PC)	To start a firm and keep it working would be easy for me.	Liñán and Chen (2009)
	I am prepared to start a viable firm.	
	I can control the creation process of a new firm.	
	I know the necessary practical details to start a firm.	
	I know how to develop an entrepreneurial project.	
	If I tried to start a firm, I would have a high probability of succeeding.	
Entrepreneurial intention (EI)	I am ready to do anything to be an entrepreneur.	Liñán and Chen (2009)
	My professional goal is to become an entrepreneur.	
	I will make every effort to start and run my own firm.	
	I am determined to create a firm in the future.	
	I have very seriously thought of starting a firm.	
	I have the firm intention to start a firm someday.	

Literature review.

Regarding the minimum sample size, the G*Power application was used considering that the snowball sampling technique is not random (Malhotra, 2011). The choice for this software was since a statistical test enables to produce a statistically significant result (Cohen, 2013). Considering that the model has four predictors, the test was performed considering an *f*^2^ of 0.15 and the number of predictors equal to three and tested for a power of 0.80 resulting in a value of 77 cases as a minimum sample. Hair et al. (2014) estimates between 2 and 3 times the ideal values. In the case between 154 and 231, since the sample has 190 respondents, it is fit for our analyses. Regarding the analyses, data were tabulated in an Excel^®^ spreadsheet and exported to IBM^®^ SPSS^®^ Statistics, version 20, for descriptive statistics calculations, and later to Smart PLS-SEM, version 3.2.9, used for structural equation modeling (Ringle et al., 2015). In addition, PLS-SEM was operated as a multiple regression analysis making it particularly valuable for exploratory research purposes, and it was employed here as it is indicated when: (a) abnormal data; (b) small samples, and (c) formative constructs (Hair et al., 2014), criteria which align with this study as well.

The choice for this statistical technique is also backed up by previous work which employed it and obtained results robust enough to prove its reliability, even to different kinds of samples, dealing with different research questions as well. Thus, is confirms its potential to answer research questions related to entrepreneurial intentions and the variables that might influence it such as gender, technology, and social capital, for instance, and it also proved its reliability in the most diverse contexts (Dana et al., 2021; Rahman et al., 2022; Ramadani et al., 2022; Salamzadeh et al., 2022).

## 4. Results

### 4.1. Sample characteristics

Table 2 shows the predominant characteristics of the sample, 57.7% of respondents are women, with the predominant age group being people from 25 to 29 years-old, represented by 24.2%. Regarding occupational status, 35.3% declared to work for a private initiative and 18.9% worked on their own, the age of respondents might be justified by the fact that the courses surveyed mainly occurred during the evening.

**TABLE 2:** Sociodemographic data.

Variable	Description	*N*	*F* %
Genre	Male	86	45,3
	Famele	104	54,7
Age range	Up to 19 years	14	7,4
	From 20 to 24 years	29	15,3
	25 to 29 years	46	24,2
	From 30 to 34 years old	39	20,5
	35 to 39 years	31	16,3
	From 40 to 44 years old	14	7,4
	From 45 years onwards	17	8,9
Educational qualification	Elementary school	2	1,1
	High school	67	35,3
	University education	55	28,9
	Post-graduate	54	28,4
	Master’s degree	11	5,8
	Doctorate degree	1	0,5
	Elementary school	2	1,1

Research data.

To verify the model, structural equation modeling was employed through the Partial Least Square (PLS) technique, using the SmartPLS 3.2.9 software. Initially, the external model reporting the relationships between constructs and indicating variables was evaluated. In this regard, reflective indicators are linked to a construct by its loads, which are the bivariate correlations between the indicator and the construct to verify their reliability and validity. The first step is to use composite reliability to assess the internal consistency of measures. Then, the second step is the validity assessment, providing convergent validity support, which occurs when each item has loads above 0.7 and when the average variance extracted from each construct is equal to or greater than 0.5.

Regarding the external loads, variables PE01 and CR05 were excluded for having a factor load below 0.70 and the variable IE04 for presenting VIF > 5 (Hair et al., 2014). Variable IE04, namely, “I am determined to create a company in the future” was also excluded for presenting VIF > 5, and PE05, i.e., “creating new solutions to problems is an important part of who I am” was removed to allow discriminant validity. Values presented in Table 3 show the compound reliability > 0.7; AVE’s > 0.5; and the values of each construct are greater than the highest square correlation with any other, confirming the model validity.

**TABLE 3:** Values of the fit quality of the external model.

Construct	SE	CR	EI	EP
Self-efficacy	**0,841**			
Creativity	0,693	**0,749**		
Entrepreneurial intentions	0,797	0,639	**0,905**	
Entrepreneurial passion	0,755	0,813	0,777	**0,794**
Cronbach’s α	0,917	0,869	0,955	0,852
Average variance extracted (AVE)	0,707	0,561	0,819	0,631

Research data. Bold values represent the *p*-values.

Table 4 below shows the values of factorial loads of observable variables (VOs) in the original constructs (VLs) are higher than in others, meeting the required criteria (Ringle et al., 2014).

**TABLE 4:** Values of the crossed loads of VOs in the VLs.

Constructs	Self-efficacy	Creativity	Entrepreneurial intentions	Entrepreneurial passion
SE01	**0,803**	0,470	0,619	0,586
SE02	**0,867**	0,583	0,706	0,634
SE03	**0,835**	0,631	0,619	0,686
SE04	**0,843**	0,506	0,578	0,538
SE05	**0,843**	0,582	0,659	0,568
SE06	**0,854**	0,684	0,800	0,758
CR01	0,565	**0,750**	0,456	0,570
CR02	0,410	**0,738**	0,366	0,596
CR03	0,480	**0,753**	0,472	0,572
CR04	0,432	**0,736**	0,414	0,601
CR05	0,594	**0,698**	0,460	0,570
CR06	0,536	**0,749**	0,544	0,617
CR07	0,584	**0,814**	0,598	0,717
EI01	0,704	0,508	**0,832**	0,631
EI02	0,724	0,596	**0,923**	0,726
EI03	0,775	0,633	**0,912**	0,726
EI04	0,749	0,625	**0,938**	0,744
EI05	0,671	0,547	**0,896**	0,669
EI06	0,695	0,547	**0,924**	0,712
EP01	0,473	0,486	0,452	**0,657**
EP02	0,713	0,680	0,752	**0,870**
EP03	0,539	0,630	0,530	**0,757**
EP04	0,570	0,586	0,614	**0,819**
EP05	0,688	0,801	0,686	**0,850**

Research data. Bold values represent the *p*-values.

According to Table 5, the values of the model’s quality of fit indicators, namely, Pearson’s coefficient of determination (*R*^2^) represent the combined effect of the exogenous variable on the endogenous ones with values of 0.75, 0.50, 0.25, respectively, describing substantial, moderate, or weak levels of predictive accuracy (Henseler et al., 2009). The predictive relevance or validity coefficient *Q*^2^ represents a means to evaluate the interior model with predictive relevance, a *Q*^2^ > 0 indicates an endogenous construction forecast, not about the quality of the forecast (Hair et al., 2014; Ringle et al., 2014). Thus, the values below show the model is internally fit and adequate for analyses.

**TABLE 5:** Predictive values.

Constructs	*R* ^2^	Adjusted *R*^2^	*Q* ^2^
Self-efficacy	0,589	0,585	0,405
Entrepreneurial intentions	0,709	0,704	0,564
Entrepreneurial passion	0,660	0,6559	0,377

Research data.

Table 6 demonstrates the model has a suitable predictive quality, being moderate in the coefficient of determination and predictive in relation to *Q*^2^. It is also possible to observe that coefficients depicting the effects between the relationships, in this case, only hypothesis H6 was not supported (*p* > 0.05).

**TABLE 6:** Structural coefficients.

	Hypotheses	Coefficient	SD	*t* Statistics	*P* Value	Supported
EP → SE	H1	0,566	0,092	6,164	0,000	YES
CR → SE	H2	0,233	0,094	2,479	0,013	YES
EP → CR	H3	0,813	0,033	24,527	0,000	YES
EP → EI	H4	0,458	0,104	4,411	0,000	YES
SE → EI	H5	0,506	0,084	6,013	0,000	YES
CR → EI	H6	−0,079	0,078	1,022	0,307	NO
**Controls**						
Gender		−0,023	0,044	0,521	0,606	NO
Age		−0,021	0,037	0,565	0,572	NO
Education		−0,074	0,044	1,701	0,082	NO

Research data.

The first hypothesis predicting the relationship between entrepreneurial passion and self-efficacy was validated (*b* = 0.566, *p* < 0.001), a result similar to the one found by Biraglia and Kadile (2017) (*b* = 0.681, *p* < 0.000) as well as Bignetti et al. (2021) (*b* = 0.266, *p* < 0.001), both validating the same hypothesis. The second hypothesis estimated the relationship between creativity and self-efficacy and was also supported (*b* = 0.233, *p* < 0.05), a result similar to one found by those who validated the same relationship (*b* = 0.304, *p* < 0.001). The third hypothesis then predicted the positive relationship between creativity and entrepreneurial passion and was also validated (*b* = 0.813, *p* > 0.001), in the same sense (Biraglia and Kadile, 2017) also confirmed the relationship between entrepreneurial passion and creativity (*b* = 0.34, *p* < 0,001).

The fourth hypothesis then assessed the relationship between entrepreneurial passion and intention and was also supported (*b* = 0.458, *p* < 0.001), a result in accordance with Neneh (2020) (*b* = 0.380, *p* < 0.01) and Bignetti et al.’s (2021) (*b* = 0.266, *p* < 0.001), who also validated the same hypothesis. Furthermore, the fifth hypothesis was validated, confirming the influence of self-efficacy on entrepreneurial intention (*b* = 0.506, *p* < 0.001), a result similar to the ones found by Shahab et al. (2019) (*b* = 0.155, *p* < 0.001) and Neneh (2020) (*b* = 0270, *p* < 0.010).

Finally, the sixth hypothesis predicted the relationship between creativity and entrepreneurial intention and was not validated (*b* = −0.079, *p* > 0.05), differently from Shahab et al.’s (2019) who identified the positive and significant effect of creativity on entrepreneurial intention (*b* = 0.211, *p* < 0.01), but in alignment with Bignetti et al.’s (2021), who also did not validate the hypothesis (*b* = −0.096, *p* > 0.05). Nevertheless, gender was not significantly directly related to entrepreneurial intentions (−0.023, *p* > 0.05), thus, there are no differences between men and women in becoming entrepreneurs in the sample analyzed in this study. Age and education were not significant as well, that is, they do not exert any impact on entrepreneurial intentions.

In this regard, this study goes into another direction when one takes into account other recent studies that examined contextual moderators, unveiling the differences between female and male respondents is contingent on different social and cultural factors. Correspondingly, these other studies observe an increase in the creativity gender gap when the country-level cultural context of the sample is communal and an elevation when it is agentic. Moreover, results also demonstrate that the gender disparity decreased as time passed, even though industry gender composition did not influence the gender gap. In this sense, the gender gap is larger when creative performance is self- versus other-reported (Hora et al., 2022).

Although this study’s results did not corroborate with the following argument, it is noteworthy that regarding gender, entrepreneurship is important as it decreases discrimination in the job market. In this regard, although Brazilian patriarchal society has roles in which women are entrenched in chauvinist social norms which may hurdle them from starting their business ventures, but even with these obstacles women end up representing nearly 80% of solo entrepreneurship, generating 9% of Brazilian gross domestic product (Ayatakshi-Endow and Steele, 2021).

Table 7 demonstrates that the multigroup analysis could not find differences between the hypotheses’ validity, differing from the total set of the sample regarding the relationship between creativity and self-efficacy, which proved to be non-significant. The difference between groups lies in the education control variable, as it was significant for men but not for women. The negative value implies that the higher the level of education, the lower the intention to become an entrepreneur.

**TABLE 7:** Multigroup analysis.

	Male			Female		
	**Coefficient**	***P* Value**	**Supported**	**Coefficient**	***P* Value**	**Supported**
EP → SE	0,457	0,025	YES	0,610	0,000	YES
CR → SE	0,360	0,080	NO	0,177	0,066	NO
CR → EP	0,829	0,000	YES	0,806	0,000	YES
EP → EI	0,409	0,048	YES	0,462	0,000	YES
SE → EI	0,539	0,000	YES	0,511	0,000	YES
CR → EI	−0,094	0,535	NO	−0,063	0,515	NO
**Controls**						
Age	−0,012	0,833	NO	−0,034	0,526	NO
Education	−0,174	0,021	YES	−0,002	0,976	NO

Research data.

## 5. Discussion

This study aimed to examine the relationship between entrepreneurial intention, self-efficacy, creativity, and entrepreneurial passion. Moreover, it had the objective to investigate the role played by creativity in the relationship between entrepreneurial intention and self-efficacy. By following these objectives, this research differed from previous studies, which dealt with business self-efficacy as an antecedent of intentions, that is, as a variable that can distinguish entrepreneurs from non-entrepreneurs (Chen et al., 1998; Drnovšek et al., 2010) or emphasized the effectiveness of self-emotional intelligence in enterprises and attitudes (Zampetakis et al., 2009).

Considering its findings, this paper’s contribution offers empirical evidence for a broader model connecting personality-related variables, such as creativity, with entrepreneurial intentions (Zhao et al., 2005). In this sense, bootstrapping results showed a significant indirect effect, according to which business self-efficacy explains the mechanism linking creativity to entrepreneurial intentions.

Accordingly, this research provides different contributions. First, it contributes to theory as it analyzes Brazil as one of the largest emerging economies in the world and, second, it enables one to understand what influences certain individuals’ intentions to start their own business ventures. Correspondingly, this research helps to advance with a research agenda that fosters the elements which have more impact on fomenting entrepreneurship in similar contexts. Moreover, it also contributes to academia as it confirms the Theory of Planned Behavior’s explanatory power and its relevance as a tool to understand the cognitive foundations of entrepreneurship. In this regard, we demonstrate that entrepreneurship is a psychosocial phenomenon, influenced by objective reasons as well as psychological elements.

Furthermore, this study contributes the literature on entrepreneurship by demonstrating the mediating roles of self-efficacy and creativity in relation to entrepreneurship and the relationship between self-efficacy and entrepreneurial passion with entrepreneurial intention. Moreover, this research introduces a model applied to a heterogeneous group composed of university students who also worked in regular jobs; thus, this sample shows a result closer to the social reality of most people in Brazil. Furthermore, this study supports the effect of self-efficacy, entrepreneurial passion, and creativity, validating a Brazilian model based on the TPB. There is also a practical contribution as this paper highlights the relevance of entrepreneurial passion and creativity in fomenting entrepreneurial intentions.

It is noteworthy to emphasize the interplay between the variables employed in this study. As highlighted in the Section “2. Theoretical backgrounds,” passion is a psychological state which can lead potential entrepreneurs to believe they are capable of undergoing certain stressful situations and still be able to reach success. In this regard, passion function as enabler entrepreneurial intention, making the individual to push certain personal and social boundaries to reach specific business goals (Shane et al., 2003; Cardon et al., 2009, 2013; Chen et al., 2009; Huyghe et al., 2016).

Correspondingly, self-efficacy intertwines with passion and intention as it is key to individuals not being strayed from their reality by passion. Self-efficacy assists the entrepreneur to see the limitations that might be found as well as the personal or organizational possibilities to overcome such limitations. Thus, this interplay has an influence on intentions as it can moderate how much one feels it can surpass an obstacle during starting as a venture but, at the same time, this person is likely to have an accurate perception of the extant of what can be actually done (Ajzen, 1991; Bandura, 2001; Schwarz et al., 2009; Drnovšek et al., 2010; Shahab et al., 2019).

In addition, the interactions between passion, self-efficacy and creativity have an influence on intention as creativity is another key trait for entrepreneurs to be successful in their endeavors, as these individuals elaborate new and resourceful ways to perform an action or solve a specific problem. In this regard, creativity makes entrepreneurs realize and develop different and potentially innovative ways, helping them to devise ways to act according to the drives of passion and self-efficacy, thus, affecting their entrepreneurial intentions as well (Young, 1985; Sternberg and Lubart, 1999; Ko and Butler, 2007; Puhakka, 2012; Smith et al., 2016).

## 6. Conclusion

Our findings point out to high values of creativity and passion (78.9%; 75.8%), with self-efficacy showing a lower value (61.4%), indicating that, although they are cognitively present as entrepreneurs, the individuals comprised in the sample are insecure about carrying out such behavior, reducing their intention to start a business (65.9%). This fact can be explained by the lack of entrepreneurial education, considering that the sample is made up of university students who did not have entrepreneurship as part of their courses’ curricula.

In addition, this research provides a contribution to both entrepreneurship theory and its cognitive foundations since it showed the impact of psychological variables like self-efficacy, entrepreneurial passion, and creativity and their influence over entrepreneurial intentions. Hence, this study contributes to the theories, entrepreneurship theory, and to the cognitive foundations of entrepreneurship by showing the importance certain psychological factors hold and their impact on entrepreneurial intentions.

Nevertheless, this research is not without limitations, the contextual specificity of the courses and the non-probabilistic sample can be cited since they make it difficult to obtain valid results for all cases. There are two possible avenues for future studies, first, to replicate the model in another context, which could be both a developing country and even in a developed one. A second avenue for future research would be to analyze the mediating relationships to expand the results and consolidate the model presented here.

The inclusion of other variables is another limitation of this work since it was not able to include other variables, such as opportunity alertness. This variable was not discussed here because it was not included in the scope of this study, which preferred to focus on self-efficacy, passion and creativity as well as to check its influence on entrepreneurial intentions. Likewise, opportunity alertness was not included in the model elaborated to answer our research questions. Accordingly, on the one hand, the hypotheses predicting the positive relationship between entrepreneurial passion and self-efficacy, creativity and self-efficacy, entrepreneurial creativity and passion, entrepreneurial passion and entrepreneurial intention, and self-efficacy and entrepreneurial intention were confirmed, on the other hand, the relationship between creativity and the entrepreneurial intention was not confirmed. Furthermore, the multigroup analysis showed that education influences men’s entrepreneurial intentions, and creativity only affects intentions when mediated by passion.

## Data availability statement

The raw data supporting the conclusions of this article will be made available by the authors, without undue reservation.

## Author contributions

MF-N focused on the search for articles, partly analyzed the material, and wrote the Portuguese version of this manuscript. JC performed part of the search for the articles, analyzed the collected material, and wrote the Portuguese version of this manuscript. JS-F was the research supervisor. MF-N, JC, and BS performed the research’s analyses and writing. BS analyzed the data collected, offered insights on new categories, and wrote the English version of this manuscript. All authors contributed to the article and approved the submitted version.
